# Study on the predictive value of APACHE II score and neurogenic dysphagia in carbapenem-resistant Klebsiella pneumoniae

**DOI:** 10.1097/MD.0000000000040858

**Published:** 2024-12-13

**Authors:** Xuan Zhou, Zhipeng Zhang, Xiaoqiong Wang, Yongsheng Wang

**Affiliations:** a Department of Pulmonary and Critical Care Medicine, The Second People’s Hospital of Hefei, Hefei Hospital Affiliated to Anhui Medical University, Hefei, Anhui Province, China; b Department of Cardiology, The Second People’s Hospital of Hefei, Hefei Hospital Affiliated to Anhui Medical University, Hefei, Anhui Province, China.

**Keywords:** APACHE II score, carbapenem-resistant Klebsiella pneumoniae, infection risk, neurogenic dysphagia, resistance

## Abstract

Carbapenem-resistant *Klebsiella pneumoniae* (CRKP) poses a growing challenge in clinical treatment globally. Early identification of high-risk patients is essential to control infection spread and improve treatment outcomes. This retrospective study analyzed 152 patients with *K pneumoniae* infections at the Second People’s Hospital of Hefei City, Anhui Province, dividing them into carbapenem-resistant and non–carbapenem-resistant groups. Clinical data, microbiological test results, Acute Physiology and Chronic Health Evaluation II (APACHE II) scores, and neurogenic dysphagia status were evaluated to identify risk factors for CRKP infection. The study revealed that patients in the carbapenem-resistant group had significantly higher APACHE II scores and a higher incidence of neurogenic dysphagia (*P* < 0.05). Multivariate logistic regression analysis identified APACHE II scores and neurogenic dysphagia as independent risk factors for CRKP infection. Receiver operating characteristic curve analysis showed an area under the curve of 0.824 (95% confidence interval: 0.749–0.898) for the APACHE II score, with an optimal threshold of 14.5 points. A new predictive model combining neurogenic dysphagia with APACHE II scores improved classification performance, as demonstrated by Net Reclassification Improvement (NRI = 0.0967, 95% confidence interval: −0.0477 to 0.2410) and reclassification probability analysis, correctly reclassifying 24.32% of individuals into a higher risk category. The findings highlight the combined predictive value of APACHE II scores and neurogenic dysphagia for early identification and intervention in high-risk CRKP patients.

## 1. Introduction

In recent years carbapenem-resistant Klebsiella pneumoniae (CRKP) has emerged as a grave challenge in global healthcare.^[1]^ This bacterium exhibits resistance to a wide range of common antibiotics resulting in poor clinical outcomes and high mortality rates. According to the latest epidemiological studies there is an alarming upward trend in the incidence of this infection. Reports indicate that the mortality rate among patients infected with CRKP can reach up to 50% a figure that is strikingly concerning.^[2]^ The occurrence of this infection is associated with various factors including a history of exposure to carbapenems nosocomial cross-infection deficits in the patient’s immune system severe underlying diseases prolonged hospitalization or undergoing invasive and invasive procedures. Therefore investigating these associated factors is crucial for designing effective prevention and control strategies.

Currently a variety of methods and indicators have been developed to predict the risk of CRKP. The Acute Physiology And Chronic Health Evaluation II (APACHE II) score a widely utilized tool for assessing the physiological status of critically ill patients has been demonstrated to be significantly valuable in predicting mixed mortality rates and clinical outcomes.^[3]^ However employing the APACHE II score to specifically predict the risk of CRKP is relatively rare in the existing literature. Central nervous system disorders affecting swallowing functions, manifesting as difficulties in swallowing and coughing while drinking, are typically observed in patients with stroke, Parkinson’s disease, cerebral hemorrhage, or extensive cerebral infarction sequelae.^[4]^ Research has shown that cricopharyngeal dysfunction following supratentorial stroke is significantly correlated with lesion location, and lesion location has a crucial impact on the incidence, pattern, and complications (such as oropharyngeal residue, swallowing and cough response, and pneumonia) of dysphagia in acute stroke patients.^[5]^ These patients often experience recurrent infections and premature exposure to carbapenems along with repeated usage leading to the emergence of resistant bacterial strains.^[6]^ Although there is a notable connection between swallowing disorders and respiratory infections the association with antibiotic resistance particularly in the context of these disorders is scarcely reported in research studies.

Therefore, the objective of this study is to evaluate the predictive value of the APACHE II score and the presence of neurogenic dysphagia in patients with carbapenem-resistant Klebsiella pneumoniae (CRKP) infections. Specifically, this study aims to determine whether these factors can serve as independent risk predictors for CRKP infections and to assess the combined predictive power of these indicators. By identifying these predictive factors, the study seeks to provide clinical insights that can aid in early identification and intervention for high-risk patients, ultimately contributing to better management and control of CRKP infections in clinical settings.

## 2. Materials and methods

### 2.1. Study design

This retrospective study was conducted at the Second People’s Hospital of Hefei, Anhui Province, a large tertiary A-level hospital. The study was approved by the Ethics Committee of the hospital, with the approval number 2023-Research-081, and was carried out in strict accordance with the Declaration of Helsinki (1964). A total of 152 patients diagnosed with Klebsiella pneumoniae infection were collected from those who visited the Department of Respiratory and Critical Care Medicine of the hospital from January 2020 to December 2022.

### 2.2. Patients

All patients met the inclusion criteria and had complete hospital records. They were divided into a non-carbapenem resistance (n = 78) and a carbapenem resistance group (n = 74) based on their susceptibility to carbapenems. The inclusion criteria for the study were as follows: aged 18 years and above; confirmed infection with Klebsiella pneumoniae through microbiological culture; the initial positive culture result was used; for patients with CRKP infections in multiple sites, only 1 incident was included. Exclusion criteria involved: incomplete record of data; hospital-acquired infection lasting <48 hours; including instances of infections involving multiple bacterial strains.

### 2.3. Microbiological examination

Microorganisms isolated were identified using traditional methods and the VITEK2 Compact® automated system (bioMérieux, Marcy l’Etoile, France). All antimicrobial susceptibility testing was performed in accordance with the 2021 guidelines of the Clinical and Laboratory Standards Institute, USA. For inclusion of CRKP, strains had to demonstrate resistance to at least one of the following antibiotics: imipenem, meropenem, ertapenem, or doripenem.

### 2.4. Date collection

Basic patient information was collected through the United Zhong Electronic Medical Record System of the hospital. This included key health indicators such as gender, age, history of neurological diseases, chronic obstructive pulmonary disease (COPD), hypertension, diabetes, and hypoalbuminemia. Additionally, data on invasive procedures during hospitalization like mechanical ventilation, intravenous catheterization, gastric and urinary catheters, and bronchoscopy were gathered. The patient’s use of antibiotics, steroids, proton pump inhibitors, and the length of hospital stay were also recorded. All patients routinely underwent a water swallow function test upon admission. The presence of a history of coughing while eating or drinking and symptoms of swallowing disorders were documented. Suspected positive cases from the initial screening underwent repeated tests at different times to confirm the diagnosis. The test results were recorded in the nursing records and medical records. Patients who exhibited symptoms identified through the water swallow test were classified as having neurogenic dysphagia.

### 2.5. Statistical analysis

Data analysis was conducted using SPSS 27.0 software and R version 4.3.1, while graphical representations were created through GraphPad Prism 9 and R version 4.3.1. Non-normally distributed quantitative data were expressed as median P50 (P25, P75) and compared between groups using the Mann–Whitney *U* test; categorical variables were represented as percentages and compared using the Chi-square test. Some continuous variables were converted into categorical ones based on optimal cutoff values determined using X-tile 3.6.1 software. A binary logistic regression model was employed to analyze the risk factors for carbapenem-resistant Klebsiella pneumoniae infection, assessing the potential synergistic effects between variables through interaction evaluation. Plot the receiver operating characteristic (ROC) curve and calculate the area under the curve (AUC). Calculate the net reclassification improvement (NRI) and integrated discrimination improvement (IDI) to assess the classification improvement of the new model. Subsequently, analyze the probability changes in reclassification. Finally, decision curve analysis (DCA) was used to evaluate the net benefit of the model at specific thresholds. A *P* value of <.05 was considered statistically significant.

### 2.6. Sample size estimation

Prior to the commencement of the study, a sample size estimation was conducted to ensure the statistical power and reliability of the study results. The calculation was performed using G*Power software, with an alpha level set at 0.05, power at 0.80, and an anticipated effect size of 0.5. Based on these parameters, the minimum required sample size was determined to be 128 patients. To account for potential data loss and incomplete records, a total of 152 patients were included in the study, exceeding the estimated minimum sample size and thereby ensuring the robustness of the study.

## 3. Results

### 3.1. Baseline data component comparison

Carbapenem-Susceptible Klebsiella pneumoniae (CSKP) group and the CRKP group indicates that there were no significant differences in baseline characteristics such as gender, age, history of COPD, hypertension, and diabetes history between the 2 groups (*P* > .05). The incidence of hypoalbuminemia was significantly higher in the CRKP group compared to the CSKP group (*P* < .05). Regarding invasive procedures during hospitalization, the proportion of patients in the CRKP group who underwent mechanical ventilation, intravenous catheterization, gastric and urinary catheterization, and bronchoscopy was significantly higher than in the CSKP group (*P* < .05). For neurogenic dysphagia, more patients in the CRKP group had a history of exposure compared to the CSKP group. Other comparisons revealed that the CRKP group had higher APACHE II scores and a greater proportion of patients with hospital stays longer than 3 weeks, with statistically significant differences (*P* < .05). There were no significant differences in the use of glucocorticoids, proton pump inhibitors, and immunosuppressants between the 2 groups (*P* > .05). For detailed information, refer to Table 1.

**Table 1:** Comparison of baseline data between the 2 groups of patients.

Characteristics	CSKP (n = 78)	CRKP (n = 74)	χ^2^	*P*
Demographic				
Sex (male)	49 (62.8)	52 (70.3)	0.945	.331
Age (yr)	67 (57, 79.25)	74 (61, 81.25)	1.924	.056
Comorbidity				
Neurogenic dysphagia	10 (12.8)	48 (64.9)	43.587	<.001
COPD	12 (15.4)	17 (23.0)	1.416	.234
Hypertension	33 (42.3)	40 (54.1)	2.099	.147
Diabetes	20 (25.6)	18 (24.3)	0.035	.851
Hypoalbuminemia	17 (21.8)	39 (52.7)	15.590	<.001
Procedures performed				
Invasive mechanical ventilation	12 (15.4)	37 (50.0)	20.829	<.001
Central venous catheterization	13 (16.7)	44 (59.5)	29.670	<.001
Nasogastric tube feeding	23 (29.5)	48 (64.9)	19.093	<.001
Urethral catheter insertion	27 (34.6)	50 (67.6)	16.496	<.001
Bronchoscopy	19 (24.4)	33 (44.6)	6.906	.009
Other				
APACHE II score	11 (12,15)	19 (17,25.25)	5.317	<.001
Hospital stay exceeding 3 wk	15 (19.2)	49 (66.2)	34.390	<.001
Glucocorticoids	22 (28.2)	22 (29.7)	0.043	.836
PPIs	33 (42.3)	36 (48.6)	0.616	.433
Immunosuppressants	14 (17.9)	24 (32.4)	3.511	.061

APACHE II = Acute Physiology and Chronic Health Evaluation II, COPD = chronic obstructive pulmonary disease, CRKP = carbapenem-resistant Klebsiella pneumonia, CSKP = Carbapenem-Susceptible Klebsiella pneumoniae, PPIs = proton pump inhibitors.

### 3.2. Logistic regression analysis of risk factors for CRKP

To minimize confounding bias, certain continuous variables were converted into categorical variables. The binary logistic regression model included variables that showed statistically significant differences in the between-group comparison, as well as some baseline data of both patient groups. The results of the univariate binary logistic regression analysis indicated that hypoalbuminemia, invasive mechanical ventilation, central venous catheterization, nasogastric tube feeding, urethral catheterization, bronchoscopy, neurogenic dysphagia, APACHE II score, and hospital stays exceeding 3 weeks were all associated with the occurrence of CRKP (*P* < .05). Subsequently, significant variables identified from the univariate analysis were collectively included in a multivariate binary logistic regression model for analysis. In our study, baseline characteristics were included in the multivariate logistic regression analysis. The rationale for this inclusion is to adjust for potential confounding factors that may affect the relationship between the primary predictors and the outcome variable. Baseline variables such as age, gender, and preexisting conditions are important confounders that can influence both the exposure and the outcome. By including these variables, we aim to obtain more accurate and unbiased estimates of the independent effects of the primary predictors. Additionally, incorporating baseline characteristics enhances the model’s fit and predictive accuracy, providing a more comprehensive understanding of the factors associated with the outcome. The results showed that neurogenic dysphagia, APACHE II score, and hospital stays exceeding 3 weeks were risk factors for the development of CRKP (*P* < .05). Refer to Table 2.

**Table 2:** Logistic regression analysis of risk factors for CRKP.

Variables	Univariate analysis		Multivariate analysis	
	OR (95%CI)	*P*	OR (95%CI)	*P*
Male	1.399 (0.710, 2.755)	.332	1.007 (0.330, 3.076)	.990
Age ≥ 65 yr	1.756 (0.892, 3.457)	.103	1.407 (0.437, 4.532)	.567
Hypertension	1.604 (0.845, 3.045)	.148	1.481 (0.521, 4.213)	.462
Diabetes	0.932 (0.447, 1.944)	.851	1.082 (0.292, 4.011)	.907
COPD	1.640 (0.723, 3.723)	.237	1.729 (0.455, 6.560)	.421
Hypoalbuminemia	3.998 (1.975, 8.093)	<.001	1.633 (0.505, 5.275)	.413
Invasive mechanical ventilation	5.500 (2.558, 11.825)	<.001	1.973 (0.537, 7.253)	.306
Central venous catheterization	7.333 (3.447, 15.602)	<.001	3.529 (0.981, 12.697)	.054
Nasogastric tube feeding	4.415 (2.233, 8.728)	<.001	1.439 (0.419, 4.947)	.564
Urethral catheter insertion	3.935 (2.005, 7.722)	<.001	1.354 (0.400, 4.581)	.626
Bronchoscopy	2.499 (1.252, 4.988)	.009	1.687 (0.482, 5.897)	.413
Neurogenic dysphagia	12.554 (5.543, 28.434)	<.001	11.804 (3.514, 39.652)	<.001
APACHE II score	3.136 (1.615, 6.088)	<.001	1.135 (1.051, 1.226)	.001
Hospital stay exceeding 3 wk	8.232 (3.924, 17.272)	<.001	4.312 (1.366, 13.614)	.013

APACHE II = Acute Physiology and Chronic Health Evaluation II, COPD = chronic obstructive pulmonary disease.

### 3.3. Subgroup analysis and interaction test

Using the APACHE II score as a key independent variable, subgroup analysis was conducted within different strata such as gender, age, and length of hospital stay, along with an interaction test. The subgroup analysis revealed that in different stratifications such as gender (male and female), age groups over 65 years, hospital stay not exceeding 3 weeks, and patients with neurogenic dysphagia, there was a significant correlation between the APACHE II score and the occurrence of CRKP (*P* < .05). In contrast, in strata such as age ≤ 65 years, hospital stay > 3 weeks, and absence of neurogenic dysphagia history, there was no significant correlation with CRKP (*P* > .05). Neurogenic dysphagia was associated with CRKP across different strata of gender, age, length of hospital stay, and APACHE II score (*P* < .05). The interaction test results showed statistical differences in the relationship between the APACHE II score and CRKP across age, length of hospital stay, and neurogenic dysphagia strata (*P* < .05), but no significant difference in the gender stratum (*P* > .05). The relationship between neurogenic dysphagia and CRKP showed differences in gender, age, hospital stay, and APACHE II score strata (*P* < .05). For detailed information, refer to Table 3.

**Table 3:** Subgroup analysis and test for interaction.

APACHE II score	OR (95%CI)	Interaction test	Neurogenic dysphagia	OR (95%CI)	Interaction test
Sex		0.305	Sex		0.009
Male	2.985 (1.325, 6.728)**		Male	12.447 (4.471, 34.650)***	
Female	3.325 (1.044, 10.585)*		Female	13.393 (3.352, 53.515)***	
Age		0.002	Age		<0.001
>65	3.009 (1.322, 6.852)**		>65	11.798 (4.384, 31.750)***	
≤65	3.102 (0.989, 9.735)		≤65	12.889 (2.964, 56.038)***	
LOS		<0.001	LOS		<0.001
>3 wk	2.175 (0.669, 7.076)		>3 wk	12.235 (2.469, 60.644)**	
≤3 wk	6.500 (2.155, 19.606)***		≤3 wk	12.222 (4.056, 36.834)***	
Neurogenic dysphagia		<0.001	APACHE II score		<0.001
Yes	5.667 (1.279, 25.114)*		>18	20.400 (5.349, 77.799)***	
No	2.071 (0.828, 5.182)		≤18	7.455 (2.420, 22.966)***	

APACHE II = Acute Physiology and Chronic Health Evaluation II, LOS = length of hospital stay.

* Indicates *P* < .05 (statistically significant).

** Indicates *P* < .01 (highly significant).

*** Indicates *P* < .001 (extremely significant).

### 3.4. Predictive value of APACHE II score and neurogenic dysphagia in the development of CRKP

**Figure 1.**
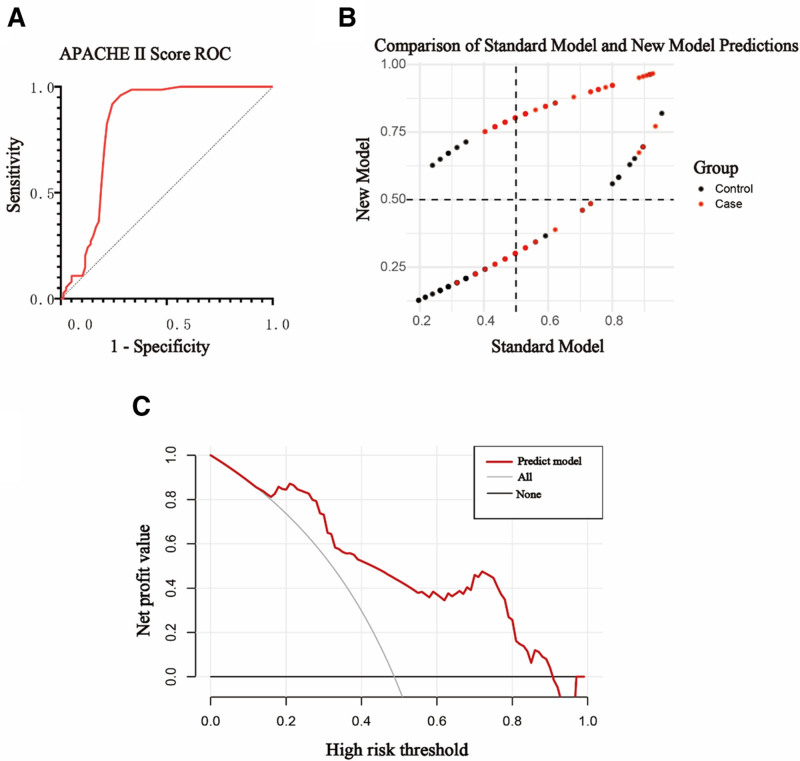
(A) Shows ROC curve for the predictive value of APACHE II score in the development of CRKP. (B) Shows scatter plot comparing the predictions of the standard model and the new model, showing the distribution of control and case groups. (C) Shows the clinical decision curve analysis for the predictive model of CRKP. From the clinical decision curve, it can be seen that within the high-risk threshold range of 0 to 0.86, the net benefit of the predictive model is positive. APACHE II = Acute Physiology and Chronic Health Evaluation II, CRKP = carbapenem-resistant Klebsiella pneumoniae, ROC = Plot the receiver operating characteristic.

The ROC curve analysis was conducted to evaluate the predictive power of the APACHE II score for CRKP. The AUC for the APACHE II score in predicting CRKP was determined to be 0.824 with a 95% confidence interval (CI) of 0.749 to 0.898, and the optimal threshold was identified as 14.5, see Figure 1A. The Hosmer–Lemeshow test indicates a good fit (χ² = 8.064, *P* = .427). Next, we incorporated neurogenic dysphagia into the APACHE II score model to predict CRKP, establishing a new predictive model. To evaluate the classification improvement of the new model, we calculated the Net Reclassification Improvement (NRI) and Integrated Discrimination Improvement (IDI). The results indicated that the new model showed a certain level of improvement in overall classification compared to the old model (NRI = 0.0967, 95% CI [−0.0526, 0.2460]). Additionally, we analyzed the probability changes of reclassification, showing that 24.32% of individuals were correctly reclassified into a higher risk category by the new model (Pr(Up|Case) = 0.2432), demonstrating good performance in identifying high-risk cases. See Table 4 and Figure 1B for details. Although the ROC curve results indicated limited improvement in classification by the new model, we retained the DCA curve analysis to further evaluate the clinical utility of the new model at different decision thresholds. See Figure 1C for details.

**Table 4:** NRI and IDI for the new predictive model.

Indicator	Estimate	Standard error	95%CI
NRI	0.0967	0.0761	[−0.0526, 0.2460]
NRI+	0.1351	0.0644	[0.0089, 0.2613]
NRI−	−0.0385	0.0421	[−0.1210, 0.0440]
Pr(Up Case)	0.2432	0.0482	[0.1480, 0.3384]
Pr(Down Case)	0.1081	0.0366	[0.0363, 0.1799]
Pr(Down Ctrl)	0.0513	0.0253	[0.0017, 0.1009]
Pr(Up Ctrl)	0.0897	0.0315	[0.0278, 0.1516]

IDI = integrated discrimination improvement, NRI = net reclassification improvement.

## 4. Discussion

With the widespread use of antibiotics, the emergence of antibiotic-resistant strains has been increasing year by year, posing a global public health challenge. Carbapenem antibiotics, commonly referred to as the “top of the antibiotic food chain,” have seen a rise in resistant strains, particularly carbapenem-resistant Enterobacteriaceae, due to their proliferation and misuse.^[7]^ A history of antibiotic usage plays a critical role in this development, as prolonged or repeated exposure to broad-spectrum antibiotics imposes selective pressure, encouraging the growth of resistant strains like CRKP.^[8]^ In particular, patients who have undergone multiple rounds of carbapenem therapy, or who are exposed to cephalosporins or fluoroquinolones before carbapenem treatment, exhibit a significantly increased risk of CRKP colonization and infection. This phenomenon is especially prevalent in intensive care units (ICUs) and long-term care facilities, where the frequent use of invasive devices further facilitates bacterial invasion and resistance spread.^[9]^ This is especially true for CRKP, where treatment options are nearly exhausted, and the strains exhibit broad resistance and high mortality rates. The Centers for Disease Control and Prevention in the United States has classified CRKP as a top “urgent threat” among antibiotic-resistant bacteria.^[10]^ Research indicates that the average time from specimen collection to effective antibiotic treatment for CRKP patients is approximately 52 hours, while the time from the onset of symptoms to death is just 96 hours.^[1]^ Therefore, conducting research on CRKP is an urgent, beneficial, and significant public health matter.

The widespread dissemination of carbapenemase genes has played a crucial role in the development of CRKP. These genes (such as KPC, NDM, VIM, IMP, and OXA-48) encode enzymes that can hydrolyze carbapenem antibiotics, rendering CRKP resistant to these last-resort antibiotics.^[11]^ This horizontal gene transfer not only facilitates the rapid spread and prevalence of CRKP but also significantly increases the difficulty of control and treatment.^[12]^ In hospital settings, especially in intensive care units and long-term care facilities, CRKP spreads more rapidly, leading to higher infection rates and posing a severe threat to patient health.^[13]^ Research has shown that carbapenemase genes can be transferred among bacteria via mobile genetic elements such as plasmids and transposons.^[14]^ These mobile genetic elements can spread not only within the same bacterial species but also across different species, allowing various bacteria to acquire resistance.^[15]^ This widespread gene dissemination further exacerbates the diversity and complexity of resistant strains. Particularly, KPC and NDM genes have attracted significant attention due to their global spread, leading to severe hospital outbreaks in multiple countries and regions.^[16]^ Additionally, the dissemination of carbapenemase genes is influenced by the use of antibiotics. Overuse and misuse of antibiotics can selectively promote the proliferation and spread of resistant strains.^[17]^ To address this challenge, comprehensive measures are needed, including strengthening antibiotic stewardship, strictly controlling infection sources, improving hospital hygiene standards, and conducting targeted research to understand the patterns of gene dissemination in different regions and environments.^[18]^

The development of CRKP is primarily attributed to the widespread dissemination of carbapenemase genes, a finding confirmed by univariate logistic regression in this study. Notably, the risk of CRKP infection is associated not only with invasive procedures such as invasive mechanical ventilation, central venous catheterization, nasogastric tube feeding, urinary catheter retention, and bronchoscopy, but also closely linked to the patient’s nutritional status. In addition, immunosuppressive agents and glucocorticoids, which were evaluated in this study, may have an impact on infection risk. Although the comparison of immunosuppressant use between the 2 groups did not reach statistical significance (*P* = .061), this result suggests a potential trend that warrants further investigation. Current research indicates that immunosuppression, whether induced by medication or underlying disease, weakens the host immune response, facilitating bacterial invasion and colonization.^[19]^ Prolonged use of glucocorticoids has also been associated with impaired mucosal barrier integrity, increasing susceptibility to infections like CRKP. These invasive procedures can damage mucosal epithelial cells, impairing the mucosal barrier function and affecting the natural defense system, leading to dysbiosis of the microbiota. This imbalance increases the likelihood of pathogen invasion and colonization, thereby elevating the risk of CRKP infection.^[8]^ Additionally, the results of this study show that patients hospitalized for more than 3 weeks have a significantly higher risk of CRKP infection (*P* < .05). This finding suggests that as hospital stays lengthen, patients may undergo more invasive procedures, which collectively increase the risk of CRKP infection. The association between prolonged hospital stays and the risk of CRKP infection has been widely reported in multiple studies. Patients with extended hospitalizations are more likely to be exposed to multidrug-resistant strains, especially in ICUs or long-term care facilities.^[20]^ This result further supports the notion that hospitalization duration is a risk factor for CRKP infection. Future studies should explore ways to optimize hospital stays and minimize unnecessary procedures to reduce the incidence of such infections.

Particularly concerning is the finding that patients with neurogenic dysphagia face a significantly higher risk of CRKP infection. Central nervous system diseases often accompany swallowing disorders, increasing the risk of aspiration, especially in patients with cerebral hemorrhage, extensive cerebral infarction, long-term bed rest, and those with a tracheostomy. Repeated infections and the recurrent or combined use of antibiotics in these patients facilitate the emergence of CRKP.^[21]^ However, the mechanisms underlying the increased risk of CRKP due to neurogenic dysphagia require further investigation and discussion to provide more effective preventive and therapeutic strategies in clinical practice. In clinical settings, strict adherence to aseptic procedures, reducing the frequency of invasive treatments, and minimizing the duration of mechanical ventilation are crucial for preventing CRKP infections.

The APACHE II score is a widely used tool for assessing the severity and prognosis of critically ill patients in intensive care.^[22]^ A high APACHE II score often reflects a more severe clinical condition in patients, with a weakened immune system making them more susceptible to bacterial infections, including those caused by drug-resistant Klebsiella pneumoniae.^[23]^ In this study, we quantified the condition of hospitalized patients infected with Klebsiella pneumoniae using the APACHE II score. At a macro level, we analyzed the likelihood of developing carbapenem antibiotic resistance in Klebsiella pneumoniae. The results showed a positive correlation between the APACHE II score and the development of resistance. This conclusion further confirms that the APACHE II score is not only an indicator for assessing patient condition but also an important tool for predicting and managing the risk of drug-resistant infections.

The subgroup analysis and interaction test results of this study demonstrate a significant correlation between APACHE II scores and the risk of CRKP infection in specific populations, particularly pronounced in patients over the age of 65, those with hospital stays of less than 3 weeks, and patients with neurogenic dysphagia. In contrast, this correlation is not apparent in younger patients, those with longer hospital stays, or without neurogenic dysphagia. The study also identifies neurogenic dysphagia as a significant risk factor for CRKP infection. Therefore, early diagnosis and individualized treatment for these high-risk groups are especially important in clinical practice.

In this study, we used logistic regression analysis and found that both the APACHE II score and neurogenic dysphagia are independent risk factors for CRKP infection. The ROC curve was plotted using the APACHE II score as a risk factor, resulting in a large area under the curve (AUC = 0.824), indicating good predictive performance. Subsequently, neurogenic dysphagia was included as another risk factor in the predictive model. The analysis showed that the model’s predictive performance was further improved (NRI = 0.0967, 95% CI [−0.0477, 0.2410]). Although the probability changes in reclassification revealed some misclassification in the model (e.g., low-risk individuals misclassified as high-risk or high-risk individuals misclassified as low-risk), the proportion of these misclassifications was relatively low. Overall, the new predictive model demonstrated better performance in correctly identifying high-risk and low-risk individuals. The results of net reclassification improvement (NRI) and integrated discrimination improvement (IDI) further validated the effectiveness of the new model. Decision curve analysis also indicated that the predictive model provides a positive net benefit for clinical decision-making within a certain range of risk thresholds. Although the ROC curve showed good predictive performance (AUC = 0.824), it has certain limitations. While AUC provides an overall measure of the model’s discriminatory ability, it does not reflect the clinical utility of specific decision thresholds. For example, choosing a high-sensitivity threshold may lead to overtreatment due to a high false-positive rate, whereas a high-specificity threshold could result in missed diagnoses of high-risk patients. Additionally, ROC analysis does not account for the imbalance between positive and negative samples, which could affect the performance of the model when applied to real-world data. To address these limitations, future research could incorporate DCA to assess the clinical benefits of the model at various thresholds and evaluate its practical utility more comprehensively.

Finally, although this study has achieved certain results in predicting the risk of CRKP, there are still some limitations that need to be considered when interpreting the results. First, the sample size is limited. Despite including multiple case and control samples, the overall sample size is still relatively small, which may affect the statistical significance and external validity of the results. Future studies should expand the sample size to improve the reliability and generalizability of the findings. Second, this study was conducted in a single medical center, which may introduce center effects and limit the generalizability of the results. Multi-center studies would help validate the applicability and widespread use of these findings. Although this study incorporated neurogenic dysphagia into the APACHE II score model, other potential predictors were not included, which may affect the predictive ability of the model. Future studies should consider more relevant variables to enhance the model’s accuracy.

## 5. Conclusion

In conclusion, both the APACHE II score and neurogenic dysphagia are independent risk factors for the occurrence of CRKP infections, and their combined predictive value surpasses that of each individual indicator, offering higher diagnostic value. Healthcare professionals should pay special attention to the APACHE II score and the history of neurological diseases when managing such patients, in order to develop more effective prevention and treatment strategies, thereby reducing the risk of CRKP infections.

## Author contributions

**Conceptualization:** Yongsheng Wang, Xiaoqiong Wang.

**Data curation:** Zhipeng Zhang.

**Formal analysis:** Zhipeng Zhang.

**Methodology:** Zhipeng Zhang.

**Software:** Zhipeng Zhang.

**Writing – original draft:** Xuan Zhou.

**Writing – review & editing:** Yongsheng Wang, Xiaoqiong Wang.

## References

[1] Lan P Jiang Y Zhou J Yu Y. A global perspective on the convergence of hypervirulence and carbapenem resistance in Klebsiella pneumoniae. J Glob Antimicrob Resist. 2021;25:26–34. 33667703 10.1016/j.jgar.2021.02.020

[2] Wang T Wang X Chen S. Emergence of colistin-heteroresistant and carbapenem-resistant hypervirulent Klebsiella pneumoniae. J Glob Antimicrob Resist. 2023;35:237–43. 37858865 10.1016/j.jgar.2023.09.020

[3] Niewiński G Starczewska M Kański A. Prognostic scoring systems for mortality in intensive care units--the APACHE model. Anaesthesiol Intensive Ther. 2014;46:46–9. 24643928 10.5603/AIT.2014.0010

[4] Kim JY Yoon SY Kim J Wook KY. Neural correlates of cricopharyngeal dysfunction after supratentorial stroke: a voxel-based lesion-symptom mapping with propensity score matched case-control. Int J Stroke. 2022;17:207–17. 33724099 10.1177/17474930211006300

[5] Suntrup-Krueger S Kemmling A Warnecke T. The impact of lesion location on dysphagia incidence, pattern and complications in acute stroke. Part 2: oropharyngeal residue, swallow and cough response, and pneumonia. Eur J Neurol. 2017;24:867–74. 28449405 10.1111/ene.13307

[6] Kazachkov M Palma JA Norcliffe-Kaufmann L. Respiratory care in familial dysautonomia: systematic review and expert consensus recommendations. Respir Med. 2018;141:37–46. 30053970 10.1016/j.rmed.2018.06.017 PMC6084453

[7] Sleiman A Fayad AGA Banna H Matar GM. Prevalence and molecular epidemiology of carbapenem-resistant Gram-negative bacilli and their resistance determinants in the Eastern Mediterranean Region over the last decade. J Glob Antimicrob Resist. 2021;25:209–21. 33812049 10.1016/j.jgar.2021.02.033

[8] De Pascale G Martucci G Montini L. Double carbapenem as a rescue strategy for the treatment of severe carbapenemase-producing Klebsiella pneumoniae infections: a two-center, matched case-control study. Crit Care. 2017;21:173. 28679413 10.1186/s13054-017-1769-z PMC5498909

[9] Fanelli C Pistidda L Terragni P Pasero D. Infection prevention and control strategies according to the type of multidrug-resistant bacteria and candida auris in intensive care units: a pragmatic resume including pathogens R0 and a cost effectiveness analysis. Antibiotics (Basel). 2024;13:789. 39200090 10.3390/antibiotics13080789 PMC11351734

[10] Centers for Disease Control and Prevention. Update on emerging infections: news from the Centers for Disease Control and Prevention. Carbapenem-resistant Klebsiella pneumoniae associated with a long-term-care facility—West Virginia, 2009-2011. Ann Emerg Med. 2012;59:434–6. 22662333 10.1016/j.annemergmed.2012.02.010

[11] Bush K. Prevalence and characterisation of carbapenemase encoding genes in multidrug-resistant Gram-negative bacilli. PLoS One. 2021;16:e0241234. 10.1371/journal.pone.0259005 PMC8559951 34723978

[12] Guo Y Liu F Zhang Y. Virulence, antimicrobial resistance, and molecular characteristics of carbapenem-resistant Klebsiella pneumoniae in a hospital in Shijiazhuang City from China. Int Microbiol. 2021;24:245–55. 10.1007/s10123-023-00357-x PMC10622345 37097488

[13] Kim S Lee H Jung M. Small wards in the ICU: a favorable measure for controlling the transmission of carbapenem-resistant Klebsiella pneumoniae. Intensive Care Med. 2021;47:310–8. 10.1007/s00134-022-06881-0 PMC9592670 36129475

[14] Wu W Feng Y Tang G Qiao F McNally A Zong Z. NDM Metallo-β-lactamases and their bacterial producers in health care settings. Clin Microbiol Rev. 2019;32:e00115–18. 30700432 10.1128/CMR.00115-18 PMC6431124

[15] Gillings MR Ghaly TM. New perspectives on mobile genetic elements: a paradigm shift for managing the antibiotic resistance crisis. Nat Rev Microbiol. 2022;20:260–75. 10.1098/rstb.2020.0462 PMC8628067 34839710

[16] Logan LK Weinstein RA. The epidemiology of carbapenem-resistant Enterobacteriaceae: the impact and evolution of a global menace. J Infect Dis. 2017;215:S28–36. 28375512 10.1093/infdis/jiw282 PMC5853342

[17] Li Y Zhang Y Sun X. National genomic epidemiology investigation revealed the spread of carbapenem-resistant Escherichia coli in healthy populations and the impact on public health. Genome Med. 2023;15:87. 38627827 10.1186/s13073-024-01310-x PMC11020349

[18] Lee SS Park SJ Chung MJ. Improved hand hygiene compliance is associated with the change of perception toward hand hygiene among medical personnel. Infect Chemother. 2020;46:165–72. 10.3947/ic.2014.46.3.165 PMC4189144 25298905

[19] Alagna L Palomba E Mangioni D. Multidrug-resistant gram-negative bacteria decolonization in immunocompromised patients: a focus on fecal microbiota transplantation. Int J Mol Sci. 2020;21:5619. 32764526 10.3390/ijms21165619 PMC7460658

[20] Zhou C Sun L Li H Huang L Liu X. Risk factors and mortality of elderly patients with hospital-acquired pneumonia of carbapenem-resistant Klebsiella pneumoniae infection. Infect Drug Resist. 2023;16:6767–79. 37881505 10.2147/IDR.S431085 PMC10595997

[21] Sasegbon A Hamdy S. The Role of the Cerebellum in Swallowing. Dysphagia. 2023;38:497–509. 33675425 10.1007/s00455-021-10271-x PMC10006062

[22] Sedloň P Kameník L Škvařil J Malý M Táborský M Zavoral M. Comparison of the accuracy and correctness of mortality estimates for intensive care unit patients in internal clinics of the Czech Republic using APACHE II, APACHE IV, SAPS 3 and MPMoIII models. Med Glas (Zenica). 2016;13:82–9. 27452324 10.17392/860-16

[23] Jia MW Liao GY Peng F Wang YL. Application of CURB-65, PSI, and APACHE II scores in the prognostic assessment of patients with severe pneumonia. J Guangzhou Med Univ. 2019;47:50–3.

